# Facile Synthesis of Smart Nanocontainers as Key Components for Construction of Self-Healing Coating with Superhydrophobic Surfaces

**DOI:** 10.1186/s11671-016-1444-3

**Published:** 2016-04-28

**Authors:** Yi Liang, MingDong Wang, Cheng Wang, Jing Feng, JianSheng Li, LianJun Wang, JiaJun Fu

**Affiliations:** Jiangsu Key Laboratory of Chemical Pollution Control and Resource Reuse, School of Environmental and Biological Engineering, Nanjing University of Science and Technology, Nanjing, 210094 People’s Republic of China; School of Chemical Engineering, Nanjing University of Science and Technology, Nanjing, 210094 People’s Republic of China

**Keywords:** SiO_2_-imidazoline nanocomposites, Acid/alkali dual-stimuli-accelerated release, Self-healing, Superhydrophobic surface

## Abstract

SiO_2_-imidazoline nanocomposites (SiO_2_-IMI) owning high loading capacity of corrosion inhibitor, 1-hexadecyl-3-methylimidazolium bromide (HMID), and a special acid/alkali dual-stimuli-accelerated release property have been synthesized via a one-step modified Stöber method. SiO_2_-IMI were uniformly distributed into the hydrophobic SiO_2_ sol to construct “host”-“guest” feedback active coating with a superhydrophobic surface (SiO_2_-IMI@SHSC) on aluminium alloy, AA2024, by dip-coating technique. SiO_2_-IMI as “guest” components have good compatibility with “host” sol-gel coating, and more importantly, once localized corrosion occurs on the surface of AA2024, SiO_2_-IMI can simultaneously respond to the increase in environmental pH around corrosive micro-cathodic regions and decrease in pH near micro-anodic regions, promptly releasing HMID to form a compact molecular film on the damaged surface, inhibiting corrosion spread and executing a self-healing function. The scanning vibrating electrode technique (SVET) was applied to illustrate the suppression process of cathodic/anodic corrosion activities. Furthermore, benefiting from the superhydrophobic surface, SiO_2_-IMI@SHSC remained its protective ability after immersion in 0.5 M NaCl solution for 35 days, which is far superior to the conventional sol-gel coating with the same coating thickness. The facile fabrication method of SiO_2_-IMI simplifies the construction procedure of SiO_2_-IMI@SHSC, which have great potential to replace non-environmental chromate conversion coatings for practical use.

**Electronic supplementary material:**

The online version of this article (doi:10.1186/s11671-016-1444-3) contains supplementary material, which is available to authorized users.

## Background

Chromate conversion coatings (CCCs) have been widely utilized as a pretreatment layer of protection of aluminium alloys due to their excellent barrier property and inherent self-healing feature [1]. However, the high toxicity and carcinogenic effects of Cr(VI) substances deviate from the concept of sustainable environmental development, and the extremely strict regulations issued by the Environment Protection Agency of governments all over the world on CCCs require researchers to develop environmentally friendly alternatives with equal or better anticorrosion performance [2]. Unfortunately, the protective abilities of various chemical conversion coating candidates, i.e. phosphate coating, rare earth conversion coating and permanganate/vanadate/tungstate conversion coatings, are still inferior to CCCs due to the lack of critical self-healing functionality. Self-healing coatings mimic the fundamental principle of living organisms, self-diagnose the internal defects or external mechanical damage and self-repair without any energy intervention to regenerate the integrity of coatings, which eliminate the potential risks, prolong the service life and achieve the long-term protection of underlying materials. Recently, Shchukin and Möhwald proposed the “host”-“guest” type of feedback active coatings (FACs), exhibiting the strong competitiveness and leading the research directions [3–7]. As a proof of conception, FACs are composed of two components: the “host” component (i.e. sol-gel coating and polymer coating) is responsible for the physical barrier, while the “guest” component, smart nanocontainers embedded in the “host” barrier coating provide an active self-healing nature. Once the aggressive species penetrate FACs through defects and initiate surface corrosion, smart nanocontainers can respond to the environmental changes around corrosive micro-regions, such as pH [8–13], electrochemical potential [14] and ionic strength [15, 16], and rapidly give the feedback, releasing entrapped corrosion inhibitors to depress corrosion spread. Organic corrosion inhibitors, including benzotriazole [17], 8-hydroxyquinoline [18] and benzimidazole [19], have been employed and successfully embedded into smart nanocontainers; once released, they can absorb on the metal surface and form a passive molecular film to retard the metal dissolution process by forming coordinate covalent bonds [20]. From the point of view of working mechanisms, the key step in the fabrication of FACs is design and fabrication of smart nanocontainers, which should possess some important characteristics, including high loading capacity of corrosion inhibitors as healing agents, good compatibility with the “host” component and a stimuli-responsive-controlled release characteristic. In general, the construction of smart nanocontainers follows two steps: (i) the scaffolds are firstly rigorously screened, which determine loading efficiency and compatibility, (ii) in order to realize stimuli-responsive release, specially, to execute zero premature release under normal conditions and release corrosion inhibitors upon external stimuli deriving from corrosive micro-regions; various exquisite gatekeepers are installed on the surface of scaffolds through various methods, including polyelectrolyte layer-by-layer technique [21–23], supramolecular assembly [24] and organofunctionalization [25]*.* Although FACs based on these smart nanocontainers demonstrate the outstanding corrosion resistance in experimental stage, the complicated routines for gatekeepers obstruct the up-scale production and the simple construction technique is attracting increasing attention and urgently needed [26–30].

Superhydrophobic surfaces, with static water contact angles higher than 150°, have attracted quite interest due to their unique water repellency and self-cleaning properties [31]. Recently, many efforts have been made to fabricate superhydrophobic surfaces on aluminium alloy substrates, which serve as a barrier layer to prevent aggressive species from reaching the surfaces of the alloy substrates and exhibit the excellent anticorrosion performance [32–34]. Herein, in accordance with the sudden changes of pH occurring in corrosive micro-regions of aluminium alloy, we introduce a facile one-step method to fabricate the novel smart nanocontainers, SiO_2_-imidazoline nanocomposites (SiO_2_-IMI), based on the modified Stöber method. On balance, SiO_2_-IMI owning high loading capacity of corrosion inhibitor, 1-hexadecyl-3-methylimidazolium (HMID), good compatibility with “host” sol-gel coating and special acid/alkali dual-stimuli-accelerated release property, are regarded as the qualified candidates for smart nanocontainers. More importantly, the easy-to-accomplish procedure is expected to promote the industrialization of FACs. SiO_2_-IMI as smart nanocontainers were incorporated into the hydrophobic sol to construct “host”-“guest” FAC with a superhydrophobic surface (SiO_2_-IMI@SHSC). Through the evaluation of electrochemical impedance spectroscopy (EIS) and scanning vibrating electrode technique (SVET), the comprehensive anticorrosion performance, and the self-healing function of multifunctional coating, SiO_2_-IMI@SHSC were systematically evaluated.

## Methods

### Materials and Instrument

tetraethoxysilane (TEOS, ≥99.0 %), HMID and 1,1,1,3,3,3-hexamethyldisilazane (HMDS) were purchased from Sigma-Aldrich. N-propanol, methanol, ethanol, n-hexane, hydrochloric acid (HCl) and ammonia solution (conc. 28 %) were of analytical grade and used without further purification. Water was purified with a Millipore Q system and had an electrical resistance of 18 MΩ cm. Transmission electron microscopy (TEM; JEM-2100, JEOL) and field emission scanning electron microscopy (FESEM; S-4800, Hitachi) were used to examine the morphology of the SiO_2_-IMI and SiO_2_-IMI@SHSC. Fourier transform infrared (FTIR) spectra were recorded on a Bruker Tensor 27 FTIR spectrometer. Scanning transmission electron microscopy (STEM) imaging and EDX mapping were recorded from FEI-Tecnai G2 F30 S-TWIN TEM operated at 200 kV. Thermogravimetric (TG) analysis was performed using a Mettler TGA/SDTA 851e instrument with a heating rate of 10 K min^−1^ under nitrogen flow. UV/Vis spectroscopy was carried out with a Shimadzu UV-1800 spectrometer. An inductively coupled plasma optical emission spectrometer (ICP-OES; PerkinElmer Optima 4300 DV) was used to monitor the amount of Si in a supernatant. The static contact angle (CA) was measured by a CA meter (XG-CAMB, XuanYi Instrument Ltd., China). The X-ray photoelectron spectra (XPS) of the coating were collected on a PHI QUANTERA II X-ray photoelectron spectrometer, using a monochromatic Al Kα radiation (*λ* = 8.4 Ǻ) as the exciting source. Atomic force microscopy (AFM; Vecco Brook DI) was used to characterize the topography of the surface of coated aluminium alloy specimens and operated in tapping mode. Raman spectroscopy was conducted using a laser confocal inVia Raman microspectrometer (Renishaw, UK) equipped with a 514-nm Ar ion solid excitation laser. Electrochemical measurements were carried out with a PARSTAT 2273 potentiostat/galvanostat (Princeton Applied Research, USA). SVET measurements were performed on equipment from Applicable Electronics Inc. (USA).

### Preparation of SiO_2_-IMI

A simple procedure described below was followed for the modified Stöber method [35]. In a typical synthesis, 10 mg HMID was added to a mixture of 80 mL ethanol with 3.0 mL ammonia solution (conc. 28 %). After stirring for 30 min, 0.1 mL TEOS was added dropwise to the solution. The molar ratio for TEOS:HMID:NH_3_·H_2_O was controlled as 17.3:1:929. The mixture was left to react under stirring at room temperature for another 24 h. The SiO_2_-IMI nanoparticles were collected by centrifugation, washed with ethanol and dried under vacuum overnight for further use.

### Preparation of SiO_2_-IMI@SHSC

The typical preparation route of SiO_2_-IMI@SHSC includes four steps: (1) formation of SiO_2_ gel; (2) modification of the surface of SiO_2_ gel with HMDS through covalently bonding with interfacial reactive –OH group to form hydrophobic SiO_2_ gel; (3) transformation of hydrophobic SiO_2_ gel to hydrophobic SiO_2_ sol with the aid of ultrasonication; (4) incorporation of SiO_2_-IMI into hydrophobic SiO_2_ sol and then to produce the novel type of “host”-“guest” FAC with a superhydrophobic surface, SiO_2_-IMI@SHSC (Fig. 4A), on AA2024 by dip-coating technique. Typically, 5.35 mL TEOS was first dissolved in 10 mL methanol, and then a mixture of 7.5 mL NH_4_OH (0.02 N) and 10 mL methanol was added to the solution. After stirring at room temperature for 2 h, 3.65 mL HCl (0.1 N) was added to the mixture. For adjusting the pH of the mixture close to 8.0, the appropriate amount of NH_4_OH solution was added. The SiO_2_ gel was formed after ageing for 20 h. Thereafter, 5 mL HMDS in 47 mL n-hexane was added to the SiO_2_ gel. After keeping at 60 °C for another 20 h in a closed container, the hydrophobic SiO_2_ gel was obtained. After that, the hydrophobic SiO_2_ gel was dispersed in 40 mL n-propanol by ultrasonication and centrifuged with a speed of 1000 rpm for 15 min to transform to the hydrophobic SiO_2_ sol. Then, SiO_2_(0.1 g) was added into 9.9 g the hydrophobic SiO_2_ sol for 5 min stirring to form a composite sol for the next dip-coating procedure.

SiO_2_-IMI@SHSC coating was prepared on an aluminium alloy surface (AA2024, 40 mm × 20 mm × 2 mm) the by dip-coating procedure. The pretreated AA2024 specimen was immersed into the composite sol for 5 min followed by withdrawal at a speed of 10 mm min^−1^ to complete the first round. The process was repeated for another three times using pure hydrophobic sol (no SiO_2_-IMI addition). The coating sample was air-dried at 120 °C for 2 h.

### Acid/Alkali Dual-Stimuli-Accelerated Release Experiments

In order to investigate the acid/alkali dual-stimuli-accelerated release characteristic of SiO_2_-IMI, UV/Vis spectroscopy was used to determine the concentration of HMID released from SiO_2_-IMI in a supernatant using the standard curve (Additional file 1: Figure S1). Briefly, SiO_2_-IMI (1 mg) were placed in the dialysis membrane at the top of the quartz cuvette to avoid interference. The solution (4 mL) of different pH values (neutral, PBS buffer solution 7.0; acidic, adjusted by HCl solution; alkaline, adjusted by NaOH solution) was carefully added into the cuvette to ensure that SiO_2_-IMI were completely immersed into the solution. Release profiles were obtained by plotting the absorption of HMID in the supernatant at *λ* = 210 nm as a function of time. In the first 4 h, the real-time monitoring of HMID was recorded at 1-s intervals.

### Electrochemical Measurements

A conventional three-electrode electrochemical cell was used, which consists of a working electrode, a saturated calomel electrode (SCE), as a reference electrode and a platinum sheet as a counter electrode. Electrochemical impedance spectroscopy (EIS) measurements were performed using a PARSTAT 2273 potentiostat/galvanostat in a frequency range from 100 kHz down to 10 mHz. The coated AA2024 specimens were used as a working electrode, and the exposed area was approximately 1.0 cm^2^. The EIS measurements were carried out at an open-circuit potential (OCP) using a sine wave of 10 mV amplitude peak to peak. The impedance data were fitted to appropriate equivalent circuits by using ZSimpWin software. For the Tafel polarization measurements, the AA2024 specimens or the coated AA2024 specimens were used as the working electrode, and the exposure area was about 0.5 cm^2^. The Tafel plots were obtained by changing the electrode potential automatically from −250 mV to +250 mV at the OCP at a scan rate of 0.166 mV s^−1^.

SVET measurements were conducted on a commercial system from Applicable Electronics controlled by ASET software. The vibrating microelectrode is a Pt-Ir microprobe with a Pt black tip (15-μm diameter), which was moved at a distance of 200 μm above the exposed surface of the tested specimens on a lattice of 21 × 21 points over an area of 4 × 4 mm^2^ (step size 200 μm). The current densities are presented in the form of 3D maps. Scans were started after 1 h immersion and were automatically collected every 2 h for the duration of the experiments. All the tests were conducted at the OCP.

## Results and Discussion

### Characterization of SiO_2_-IMI

**Fig. 1:**
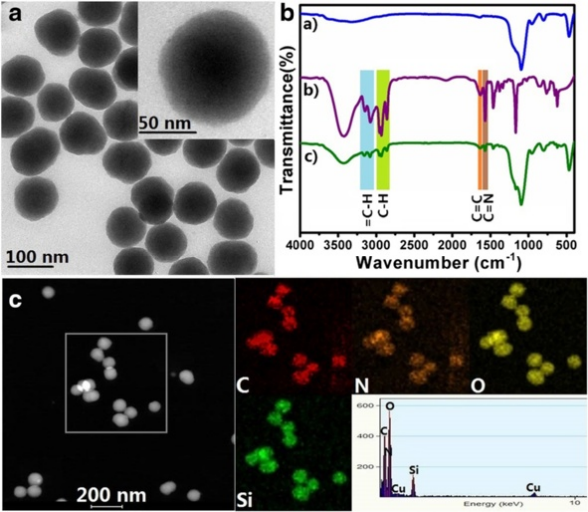
**a** TEM image of SiO_2_-IMI. **b** FTIR spectra of the bare SiO_2_ nanoparticles (*a*), HMID molecule (*b*) and SiO_2_-IMI (*c*). **c** STEM image and EDS mapping of SiO_2_-IMI

**Fig. 2:**
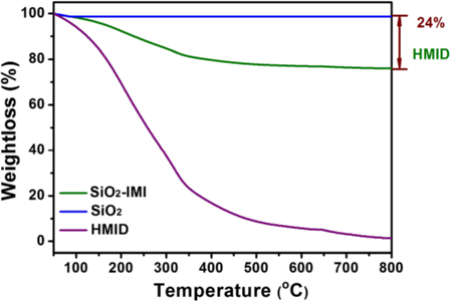
TG analysis of SiO_2_-IMI

The synthetic route of the smart nanocontainers, SiO_2_-imidazoline nanocomposites (SiO_2_-IMI), is based on the modified Stöber method. SiO_2_-IMI were formed by using tetraethoxysilane (TEOS) as a silica source and aqueous ammonium as a catalyst in the presence of an imidazoline derivative, HMID, as corrosion inhibitor. The morphology of SiO_2_-IMI was characterized by TEM and FESEM. As displayed in Fig. 1a and Additional file 1: Figure S2, the nearly monodispersed spherical SiO_2_-IMI with a uniform diameter of about 100 nm were successfully obtained. The implantation of HMID molecules into the silica matrix was confirmed by FTIR spectroscopy and STEM coupled with energy-dispersive spectroscopy (STEM-EDS). The FTIR spectra of the bare SiO_2_ nanoparticles, HMID molecule and SiO_2_-IMI are shown in Fig. 1b. After comparison, in addition to the typical bands at 1097, 930 and 798 cm^−1^ belonging to Si-O-Si network vibration, the absorption bands at 2931 and 2860 cm^−1^, corresponding to C-H stretching vibrations, and the characteristic peaks at 1633 (C=C stretching vibration), 1571 (C=N stretching vibration) and 3076 and 3144 cm^−1^ (unsaturated C-H stretching vibration), assigning to the imidazoline group, all demonstrate the existence of HMID components in SiO_2_-IMI. The STEM image and EDS mapping of SiO_2_-IMI in Fig. 1c exhibit the definite distribution of elements C and N within SiO_2_-IMI nanocomposites, which originate from an HMID molecule. Thermogravimetric (TG) analysis provides the rough estimation of HMID content of SiO_2_-IMI as 240 mg HMID g^−1^ SiO_2_-IMI (Fig. 2), which exceeds the adsorbed amount of the corrosion inhibitor in mesoporous materials as scaffolds and meets the requirement of smart nanocontainers for self-healing agents.

### Acid/Alkali Dual-Stimuli-Accelerated Release of HMID from SiO_2_-IMI

**Fig. 3:**
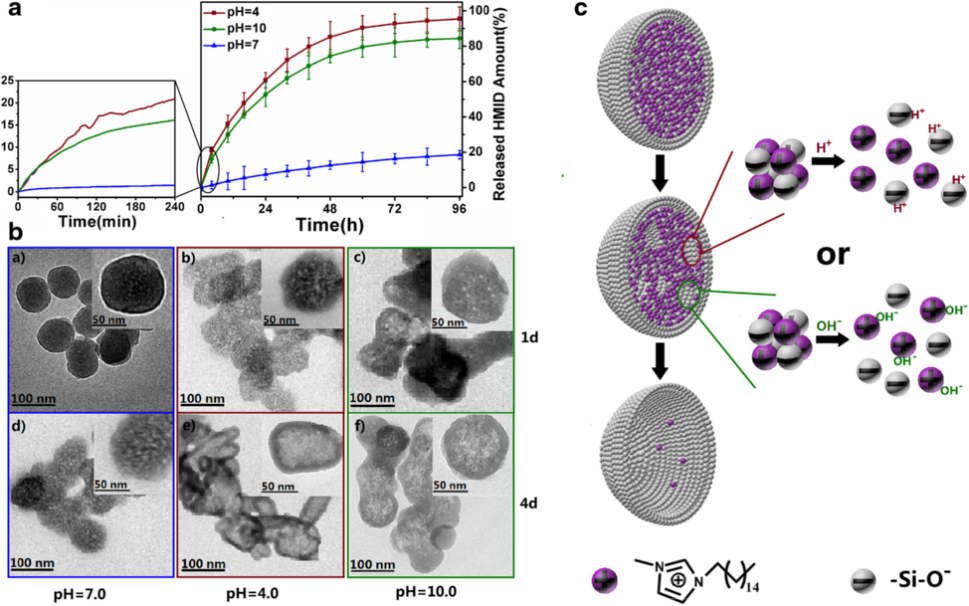
**a** Release profiles of HMID from SiO_2_-IMI at different pH values. **b** TEM images of the SiO_2_-IMI taken after being immersed in solution with different pH values (pH 7.0 (*a*, *d*); pH 4.0 (*b*, *e*); pH 10.0 (*c*, *f*)) for 1 day (*a*, *b*, *c*) and 4 days (*d*, *e*, *f*). **c** The acid/alkali stimuli-accelerated release mode of SiO_2_-IMI

The “host”-“guest” FACs as a pretreatment layer deposited on the aluminium alloy are especially vulnerable to localized corrosion attack due to its relative thin thickness. The corrosive species such as Cl^−^, SO_4_^2−^ or O_2_ are prone to intrude into the coating, reach the surface and initiate the localized corrosion. In the meantime, pH values around micro-anodic regions always decrease to the acidic range due to the dissolution of Al and subsequent hydrolysis of Al^3+^, whereas in micro-cathodic regions, the local alkalization regions are concurrently formed. Therefore, the pH changes are commonly considered as reliable triggers to open smart nanocontainers to release entrapped corrosion inhibitor and implement auto-repairing process [36, 37]. To investigate the stimuli-responsive characteristics of SiO_2_-IMI and ascertain their suitability for smart nanocontainers, release experiments were carried out under prespecified pH values (Fig. 3a). In the neutral solution, the negligible HMID release (less than 1.5 %) was observed within 4 h, after continuous monitoring for 4 days, about 17 % HMID was detected in the supernatant. It is worthwhile to note that the release profiles of HMID from SiO_2_-IMI under acidic or alkaline conditions are different from those under the neutral solution. The initial release rates dramatically increased, and approximately 21 and 16 % of HMID were released within 4 h under pH 4.0 and pH 10.0, respectively. At the end of the release experiment, nearly 95 % embedded HMID was released under pH 4.0, which was slightly more than that under pH 10.0. Furthermore, the pH-dependent release rate was found, the stronger acidity or alkalinity was applied and the faster release rate was obtained (Additional file 1: Figure S3 and Table S1). It is not difficult to analyse that the acid/alkali dual-stimuli-accelerated release characteristic qualifies SiO_2_-IMI as the ideal smart nanocontainers. No obvious premature release of HMID in a short period of time under normal circumstances ensure the stable and effective content of self-healing agents before working in corrosive micro-regions. Meanwhile, SiO_2_-IMI can automatically feel the pH stimuli occurring in either the micro-anodic or micro-cathodic regions, releasing HMID to give a feedback without delay. The release amount of HMID from SiO_2_-IMI in the first 4 h under pH 4.0 and pH 10.0 is, respectively, 14 and 11 times larger than that under pH 7.0. Obviously, the initial quick releasing of HMID triggered by acid or alkali stimuli are helpful to possible self-healing process. Furthermore, the simple preparation method has a potential for practical application. Different from the two conventional strategies for accommodation cargoes in silica materials, i.e. adsorption of cargoes in mesoporous silica materials and construction of cleavable chemical linkage between cargoes and silica materials, the growth pattern of SiO_2_-IMI is in a special way, which is the crucial point to realize acid/alkali dual-stimuli-accelerated release. The TEM images taken at different stages of release experiments have contributed to investigate the working mechanisms of SiO_2_-IMI. After release for 1 day in neutral solution, SiO_2_-IMI did not greatly change in morphology; however, a few small holes apparently emerged in the interior region at the end of the release experiment (Fig. 3b (*a*, *d*)). In contrast, SiO_2_-IMI appeared to have changed considerably, and the clear hollow structure was observed after 4 days release under pH 4.0 (Fig. 3b (*b*, *e*)). The ICP-OES was adopted to monitor the amount of Si in the supernatant, which shows the increase trend with the elapse of time (Table 1). As for the alkali stimuli (pH 10.0), the complete dissolution phenomenon of SiO_2_-IMI was not found; instead, the self-degradation mainly happening at the interior region was pronounced, and the exterior layer was relatively robust (Fig. 3b (*c*, *f*)).

**Table 1:** Amount of Si in the supernatant during release experiments

Time (days)	pH values	Amount of Si (mg/L)
1	7.0	0.41
	4.0	3.34
	10.0	2.89
4	7.0	2.04
	4.0	10.46
	10.0	9.63

Based on the Stöber method, SiO_2_-IMI was successfully synthesized by introducing appropriate amounts of HMID at the beginning stage of reaction and precisely controlling reaction conditions. During the process of ammonia-catalysed hydrolysis and condensation of TEOS, the silica species carry high negatively charged density under alkaline condition and are apt to bind with positively charged HMID molecules via strong electrostatic attraction. Due to the high initial concentration of HMID, the composite condensation product quickly exceeds the critical saturation, and a large amount of stable silica-HMID nuclei are formed and further aggregated. As the reaction proceeded, the massive consumption makes the shortage of HMID in the later SiO_2_-IMI growth stage. The specific “interior-rich and exterior-deficient” distribution framework for HMID dominates the acid/alkali dual-stimuli-accelerated release. SiO_2_-IMI showed the good stability in neutral solution within a certain time; however, the slight degradation phenomenon appeared for longer time observation, which also had been reported in some previous literatures [38]. The experimental data verify that the presence of H^+^ and OH^−^ evidently accelerate the self-degradation rate. Combined with the representative TEM images, the persistent infiltration of H^+^ and OH^−^ through open pores stemming from particles packing undoubtedly destroyed the original charge balance of silica-HMID nuclei, the abrupt disappearance of strong electrostatic attraction resulted in the fact that the more aggregations of silica-HMID nuclei dissociated from SiO_2_-IMI and dispersed in the supernatant [39, 40]. The nonuniform distribution of silica-HMID nuclei determines disintegration mode of “from interior to exterior” [41]. Under a H^+^ or OH^−^ attack, the central interior regions completely collapsed and the hollow structure emerged. The outmost layer of SiO_2_-IMI retained the intact state due to its stable silica constituents and lack of silica-HMID nuclei, confirming our hypothesis for growth mechanism of SiO_2_-IMI. In addition, the morphology of SiO_2_-IMI after 4 days of immersion under pH 4.0 changed greatly (Fig. 3b (*e*)). The fast dissolution of silica-HMID nuclei within the interior regions may result in the irregular morphology. The acid/alkali stimuli-accelerated release mode is depicted in Fig. 3c.

### Construction of Multifunctional Coating, SiO_2_-IMI@SHSC

**Fig. 4:**
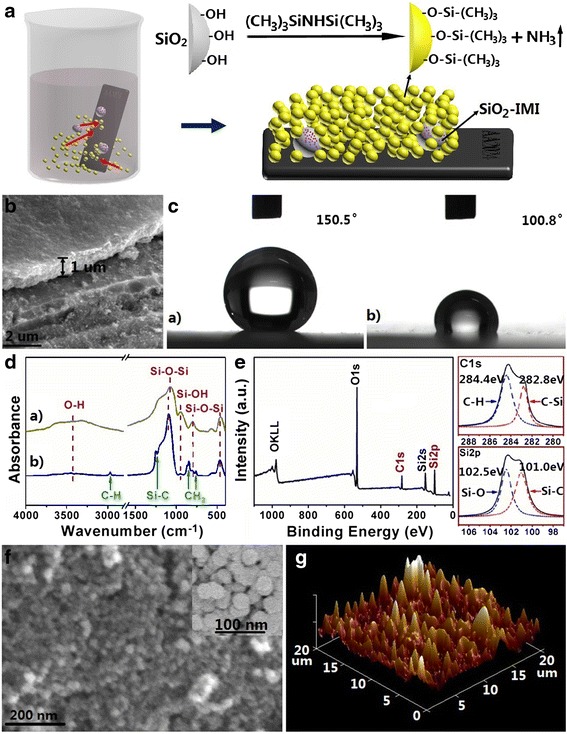
**a** Preparation route of SiO_2_-IMI@SHSC. **b** SEM cross section image of SiO_2_-IMI@SHSC. **c** CA image of SiO_2_-IMI@SHSC (*a*) and SC (*b*). **d** ATR-FTIR spectra of SC (*a*) and SiO_2_-IMI SHSC (*b*). **e** Wide-scan XPS spectrum, XPS high-resolution C1s core line spectrum and N1s core line spectrum of SiO_2_-IMI@SHSC. **f** FESEM and **g** AFM images of the surface of SiO_2_-IMI@SHSC

In order to achieve the better compatibility with the “guest” component, SiO_2_-IMI, and provide the more excellent penetration resistance towards water, hydrophobic SiO_2_ sol-gel coating was selected as the “host” component. The typical preparation route of SiO_2_-IMI@SHSC is illustrated in Fig. 4a. During the dip-coating procedure, the presetting down- and up-process were repeated for four times to increase coating thickness to about 1.0 μm, which was visualized by a cross-sectional SEM image (Fig. 4b). It needs to emphasize that the hydrophobic SiO_2_ sol doped with SiO_2_-IMI was only used for the first round dipping to concentrate SiO_2_-IMI near the surface of AA2024, which is expected to improve the working efficiency of SiO_2_-IMI. As shown in Fig. 4c, SiO_2_-IMI@SHSC presents a superhydrophobic surface with a water CA 150.5°, which is far greater than that of the SiO_2_ sol-gel coating (SC; see supporting information for preparation, no HMDS modification, no SiO_2_-IMI incorporation). ATR-FTIR and XPS were used to characterize the surface chemical composition. Compared with the ATR-FTIR spectrum of SC, the additional absorption peaks at 2964 cm^−1^ (C-H stretching vibration) and 879, 847 and 758 cm^−1^ (C-H rocking vibration) in the spectrum of SiO_2_-IMI@SHSC indicate the existence of the interfacial methyl group (Fig. 4d). Figure 4e displays the XPS spectrum of the SiO_2_-IMI@SHSC surface. The presence of C 1s, O 1s and Si 2p signals was found and no trace of N element, suggesting the decomposition of the secondary amine group of HMDS and the absence of SiO_2_-IMI in the upper coating. The high resolution of the C 1s spectrum consists two peaks corresponding to –C–Si– (located at 282.8 eV) and –C–H (located at 284.4 eV). Similarly, the Si 2p peak can also be resolved into two components assigned to –Si–O– (located at 102.5 eV) and –Si–C– (located at 101.0 eV). All of these data confirm that the coverage of hydrophobic –Si–(CH_3_)_3_ groups is the major reason for a superhydrophobic surface (Fig. 4a). Another decisive factor is the surface topography, which was illustrated by FESEM and AFM. The random accumulated hydrophobic SiO_2_ nanoparticles (*ca.* 30-nm average diameter) shown in Fig. 4f increase the root-mean-squared roughness to a certain extent, which is calculated as 51 nm by AFM (Fig. 4g). The micro/nanostructured surface topography cooperated with the low surface energy materials conform to the conventional construction method for superhydrophobic surfaces [42].

### Comprehensive Anticorrosion Performance Evaluated by EIS

**Fig. 5:**
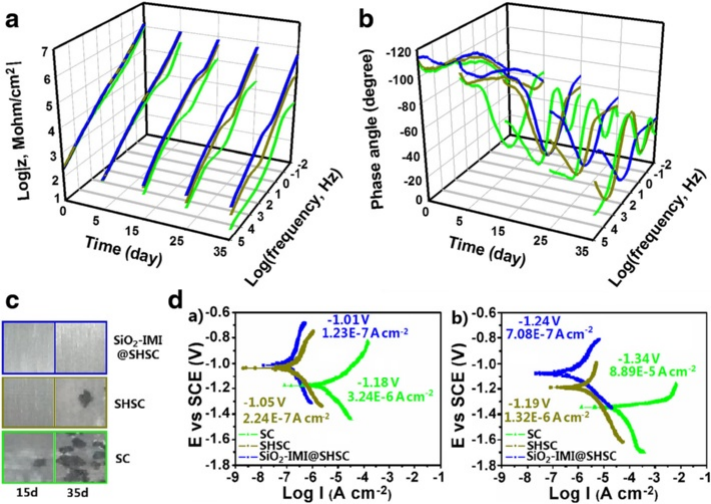
**a**, **b** Bode plots obtained on SC (*green*), SHSC (*dark yellow*) and SiO_2_-IMI@SHSC (*blue*) after immersion for 0, 5, 15, 25 and 35 days in 0.5 M NaCl. **c** Optical photos of SC, SHSC and SiO_2_-IMI@SHSC taken after 15 and 35 days of immersion in 0.5 M NaCl. **d** Potentiodynamic polarization curves of SC, SHSC and SiO_2_-IMI@SHSC at the beginning (*a*) and the 15th day (*b*) of immersion

The anticorrosion performances of AA2024 coated with SiO_2_-IMI@SHSC were evaluated by EIS, and SC and hydrophobic SiO_2_ sol-gel coating (SHSC; no addition of SiO_2_-IMI; see supporting information for preparation) were prepared as the reference coatings for comparison. Figure 5a, b shows the evolutions of the Bode spectra of the three investigated coatings with time in 0.5 M NaCl. At the beginning of immersion, the phase angle Bode diagram was very broad and only one time constant associated with physical barrier coating response was observed. Almost identical slopes reflected as a pure capacitive behaviour in the impedance frequency Bode diagram indicate that the three coatings have the same thickness, which can be corroborated by the SEM images (Additional file 1: Figure S4) and considered as a prerequisite for comparison. After 5 days of immersion, the Bode plots of SHSC and SiO_2_-IMI@SHSC remained substantially unchanged, whereas the second time constant in the medium frequency range 10^0^–10^2^ Hz associated with the response of aluminium oxide layer appeared in the plot of SC, accompanied by the decrease of impedance modulus at the lowest frequency, |Z|_0.01 Hz_. As the immersion went on to 15th day, the degradation of SC, manifested by the low phase angle at the highest frequency, was quite clear, and the third time constant in the low frequency range 10^−2^–10^0^ related to the charge transfer resistance occurring at the metal/solution interface was detected, which was evidenced by the appearance of some pitting holes on the surface (Fig. 5c). As a contrast, SHSC and SiO_2_-IMI@SHSC displayed the only minor degradation of physical barrier coatings and no visible signs of pitting corrosion, which could be unquestionably attributed to the superhydrophobic surface. A superhydrophobic surface with water repellency property is an efficient technique to prevent corrosion. According to the Cassie-Baxter model, air pockets trapped in the gaps originated from the micro/nanohierarchical surface structure make water droplets suspended on the top and thus minimize the contact area between the water containing aggressive species and the surface, enhancing the protection performance by retarding the invasion speed through the main body of coatings [43–45]. The Tafel polarization technique was employed to further inspect the effectiveness and durability of superhydrophobic surfaces. Through comparing the electrochemical parameters, *I*_corr_ and *E*_corr_, extrapolated from Tafel polarization segments, the expectant orders of *I*_corr, SHSC_ ≈ *I*_corr, SiO2-IMI@SHSC_ < *I*_corr, SC_ and *E*_corr, SHSC_ > *E*_corr, SiO2-IMI@SHSC_ > *E*_corr, SC_ measured at the beginning and the 15th day immersion reconfirm the anticorrosive advantages of superhydrophobic surfaces (Fig. 5d). On the 25th day, there was obvious difference of resistive plateau at 10^1^–10^3^ Hz between SHSC and SiO_2_-IMI@SHSC. Due to SiO_2_-IMI as nanofillers decreasing the coating porosity and delaying the diffusion rate of aggressive species, the anti-penetration ability of SiO_2_-IMI@SHSC is superior to SHSC. Until the end of the immersion for 35 days, SC completely lost its protective function and the aluminium alloy surface suffered from the severe corrosion. As for SHSC, the third time constant was more evident and the black pitting corrosion spots were macroscopically observed (Fig. 5c). Only SiO_2_-IMI@SHSC preserved the intact defence capability. The highest value of |Z|_0.01 Hz_ is the integrated result of a satisfactory barrier property and potential active protection. The difference in the protective effect between SHSC and SiO_2_-IMI@SHSC is attributed to the incorporated SiO_2_-IMI. Except for the nanofillers’ roles in SiO_2_-IMI, the responsive release of HMID from SiO_2_-IMI maybe another factor for surprising anticorrosion performance.

**Fig. 6:**
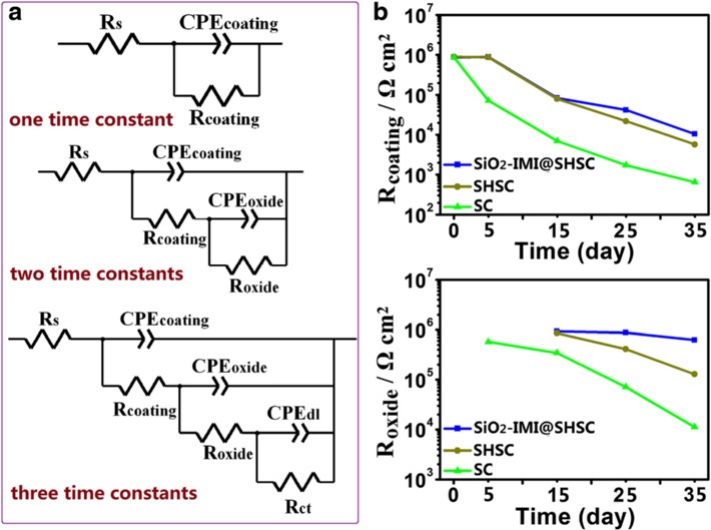
**a** The equivalent circuit models and **b** evolution of *R*_coating_ and *R*_oxide_ obtained by fitting Bode plots using appropriate equivalent circuits

The impedance data were quantitatively interpreted by equivalent electrical circuits shown in Fig. 6a. The appropriate equivalent electrical circuit was chosen to fit according to the numbers of time constants. In these equivalent electrical circuits, *R*_s_, *R*_coating_, *R*_oxide_ and *R*_ct_ represent the electrolyte resistance, pore resistance of coatings, aluminium oxide layer resistance and charge transfer resistance, respectively. Each resistance is in parallel with a corresponding constant phase element (CPE) accounting for the coating capacitance (CPE_coating_), the aluminium oxide layer capacitance (CPE_oxide_) and the charge transfer capacitance (CPE_dl_). The evolution of *R*_coating_ shown in Fig. 6b reveals the deterioration trend of physical barrier coating. The continuous decrease of *R*_coating_ for SC was caused by electrolyte uptake. Benefiting to superhydrophobic surfaces, the dropping trends of *R*_coating_ for SHSC and SiO_2_-IMI@SHSC are slower than that of SC, suggesting the excellent isolation performance. *R*_ct_ describes progress of electrochemical reactions at aluminium alloy/solution interface [46–48]. Once aggressive species pierce through physical coating, the thin and porous aluminium oxide layer cannot remain as a reliable long-term protection; the fast drop of *R*_ct_ for SHSC from the 15th day to the 35th day strengthens the above opinion. During the same period of immersion, the *R*_ct_ values of SiO_2_-IMI@SHSC did not fluctuate widely. The active actions of releasing corrosion inhibitors in a local area triggered by corrosive environmental stimuli can effectively compensate defects of oxide layer and keep the last dense line to resist corrosion.

### Self-Healing Function Evaluated by SVET

**Fig. 7:**
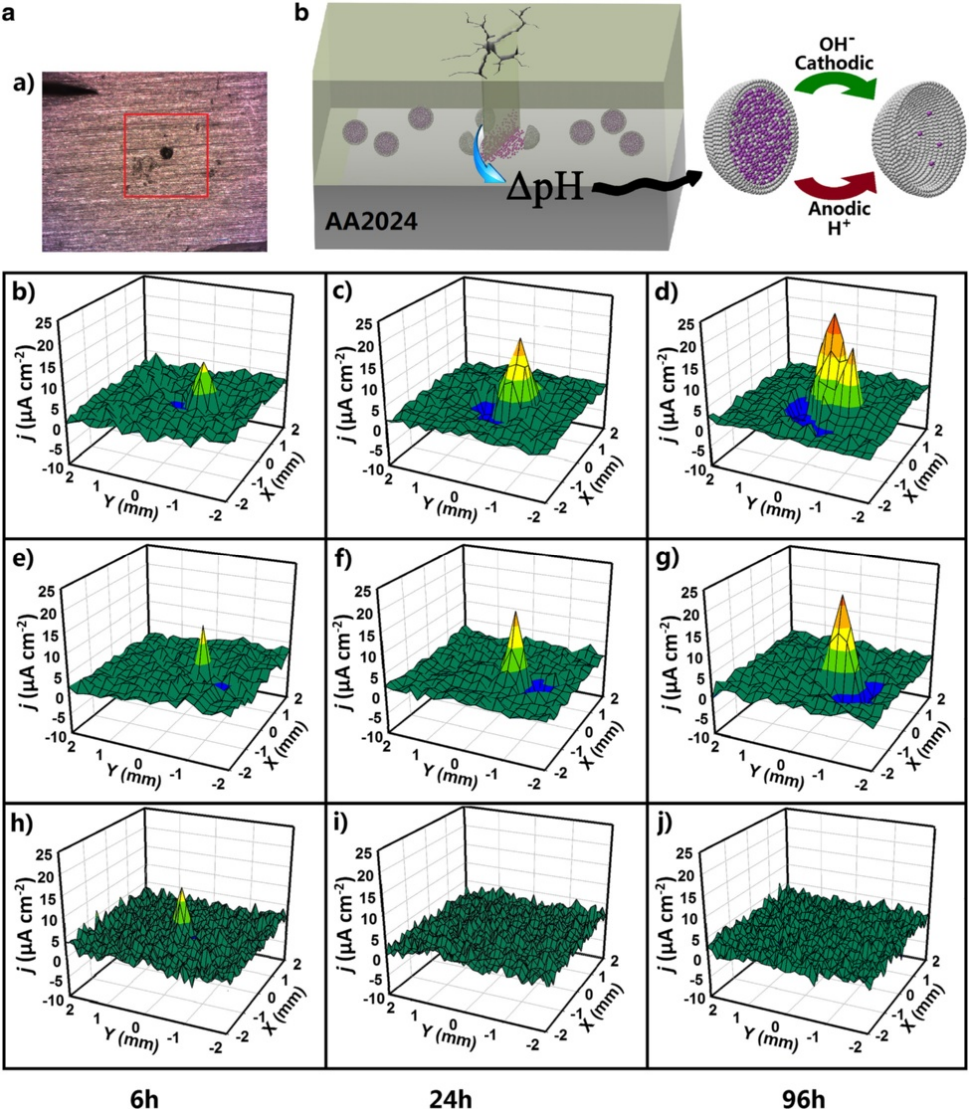
**a** Optical micrograph of the artificial defects on the coated AA2024 specimens (*a*) and SVET maps of the ionic currents measured above the defected surface of SC (*b*–*d*), SHSC (*e*–*g*) and SiO_2_-IMI@SHSC (*h*–*j*) at different stages after immersion in 0.1 M NaCl. **b** Self-healing mechanism of SiO_2_-IMI@SHSC

The self-healing function of SiO_2_-IMI@SHSC coating was graphically evaluated by SVET. The artificial dot defects (around 200-μm diameter and deep to the AA2024 surface) were carefully made on the three investigated coatings by a needle and immersed in 0.1 M NaCl solution. The evolution of ionic flux signals around artificial defects were recorded, transformed to current density data and shown in Fig. 7 as 3D maps. SC and SHSC revealed the similar trend that the anodic current density significantly increased and the weak cathodic current area gradually expanded with time. At the end of immersion for 96 h, the magnitude of anodic current peak located in the central of the scratched region reached 18.7 and 14.8 μA cm^−2^ for SC and SHSC, respectively. In stark contrast, SiO_2_-IMI@SHSC illustrated the exciting auto-suppression of local corrosion activities. The anodic and cathodic current densities emerged on the 6 h of immersion were subsequently inhibited. During the next monitoring period, no noticeable anodic or cathodic current density was measured, suggesting that the designed self-healing function operated normally. In the view of working mechanism, the corrosive species can easily permeate into the bare aluminium alloy surface along the artificial defects and initiate the localized corrosion, and then the pH around the corrosive micro-regions changed abruptly. SiO_2_-IMI near the defects aggregate and experience the increase or decrease in pH. Wherever situated, SiO_2_-IMI possessing the acid/alkali dual-stimuli-accelerated release character guarantees the prompt and sustained release of the corrosion inhibitor, HMID, which has been proved that it acts as the mix-type corrosion inhibitor and shows the excellent inhibition performance for aluminium alloy in saline solution (Additional file 1: Figure S5). It is conceivable that the local high concentration of HMID facilitates formation of molecular film on the damaged aluminium alloy surface via physisorption and chemisorption, separating aggressive species from metal surface and stopping corrosion propagation. In order to prove our hypothesis, the HMID molecular film on the artificial scratch of SiO_2_-IMI@SHSC was detected by a laser confocal micro-Raman spectrometer (Additional file 1: Figure S6). SC and SHSC without dopant of smart nanocontainers are not capable for executing self-diagnosis and self-healing tasks, which cause the more and more serious corrosion expansion.

## Conclusions

In summary, a facile approach to acid/alkali dual-stimuli-accelerated release system, SiO_2_-IMI, was demonstrated. Considering the high content of corrosion inhibitors, SiO_2_-IMI have the potential to be ideal smart nanocontainers for “host”-“guest” FACs to execute self-healing tasks in the corrosive micro-regions upon pH stimuli. The sol-gel coating with superhydrophobic surfaces was constructed as a “host” barrier coating component, and the good compatibility between the “host” coating and “guest” SiO_2_-IMI makes the fabricated multifunctional coating, SiO_2_-IMI@SHSC, work normally. SiO_2_-IMI@SHSC exhibited the excellent long-term anticorrosion performance in 0.5 M NaCl depending on the superhydrophobic, water-repellent surface and active corrosion protection from the incorporated SiO_2_-IMI. When SiO_2_-IMI@SHSC was mechanically scratched, SiO_2_-IMI were in response to corrosive environmental stimuli and released embedded HMID molecules to form protective molecular film and provide the reliable protection for a damaged metal surface. The outstanding comprehensive anticorrosion capacity and the simple preparation technique will make the multifunctional coating become a promising candidate to replace non-environmental chromate conversion coatings for the protection of aluminium alloys.

## Additional File

Additional file 1: Supporting information associated with this article, including, the preparation of SC and SHSC, standard curve of HMID, FESEM image of SiO_2_-IMI, release profiles of HMID from SiO_2_-IMI under different pH values, cross section SEM images of SC and SHSC, the Tafel plots and Laser Micro-Raman spectra, can be found in the online version at http://dx.doi.org/. (XLS 75.0 kb)
