# Supplementary material for: Facile Synthesis of Smart Nanocontainers as Key Components for Construction of Self-Healing Coating with Superhydrophobic Surfaces
Source: Nanoscale Res Lett. 2016 Apr 28;11:231. doi: 10.1186/s11671-016-1444-3 (PMC4848275; doi:10.1186/s11671-016-1444-3)
Supplement: Additional file 1: — Supporting information associated with this article, including, the preparation of SC and SHSC, standard curve of HMID, FESEM image of SiO2-IMI, release profiles of HMID from SiO2-IMI under different pH values, cross section SEM images of SC and SHSC, the Tafel plots and Laser Micro-Raman spectra, can be found in the online version at http://dx.doi.org/. (XLS 75.0 kb) [file 11671_2016_1444_MOESM1_ESM.docx]

Supporting information

Facile Synthesis of Smart Nanocontainers as Key Components for Construction of Self-Healing Coating with Superhydrophobic Surface

Yi Liang^a,1^, MingDong Wang^b,1^, Cheng Wang^b,1^ , Jing Feng^b^, JianSheng Li^a^, LianJun Wang^a^, JiaJun Fu^a,b,^*

^a^Jiangsu Key Laboratory of Chemical Pollution Control and Resource Reuse, School of Environmental and Biological Engineering, Nanjing University of Science and Technology, Nanjing, 210094, P. R. China

^b^School of Chemical Engineering, Nanjing University of Science and Technology, Nanjing, 210094, P. R. China

^1^MingDong Wang and Yi Liang contributed equally to this work

E-mail: [fujiajun668@gmail.com](mailto:fujiajun668@gmail.com)

**Table of Contents**

[1 Preparation of SHSC coating and SC coating 2](#_Toc443307645)

[2 Analytical data 2](#_Toc443307646)

[2.1 Standard curve 2](#_Toc443307647)

[2.2 FESEM image 2](#_Toc443307648)

[2.3 Release profiles of HMID from SiO_2_–IMI under different pH values 3](#_Toc443307649)

[2.4 SEM and CA images 3](#_Toc443307650)

[2.5 Tafel plots 4](#_Toc443307651)

[2.6 Laser Micro-Raman spectra 5](#_Toc443307652)

# Preparation of SHSC coating and SC coating

The only difference between the preparation route of SHSC and SiO_2_-IMI@SHSC is no SiO_2_-IMI addition in hydrophobic sol. As for SC coating, compared with SiO_2_-IMI@SHSC coating, no HMDS was modified and no SiO_2_-IMI was added during the preparation procedure. The dip-coating procedure was repeated four times to keep the similar coating thickness as SiO_2_-IMI@SHSC.

# Analytical data

### 2.1 Standard curve


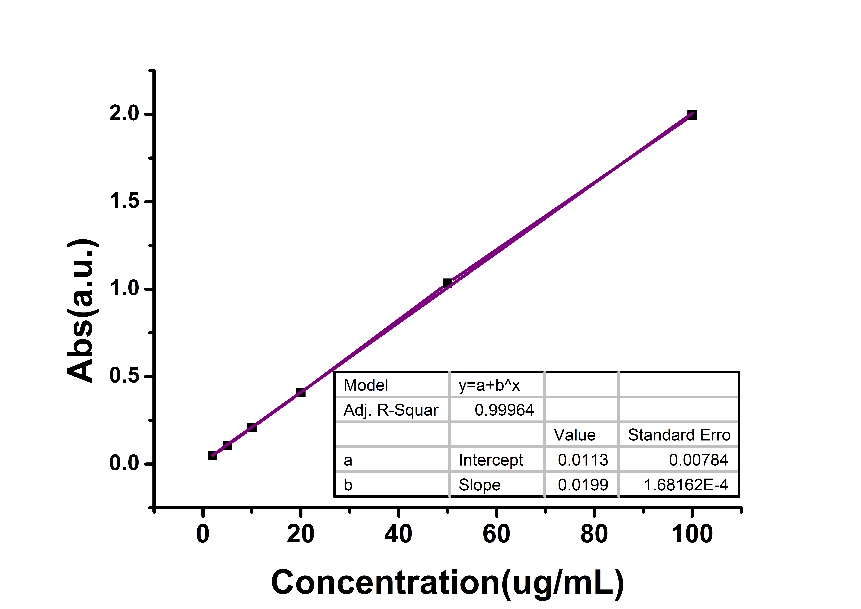

**Fig. S1.** Standard curve of UV-Vis absorption intensity of HMID.

### 2.2 FESEM image


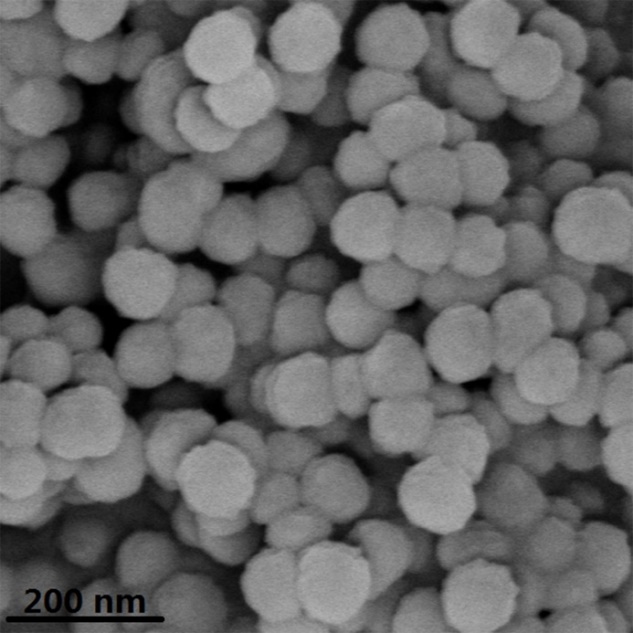


**Fig. S2.** FESEM image of SiO_2_-IMI.

### 2.3 Release profiles of HMID from SiO_2_–IMI under different pH values

**
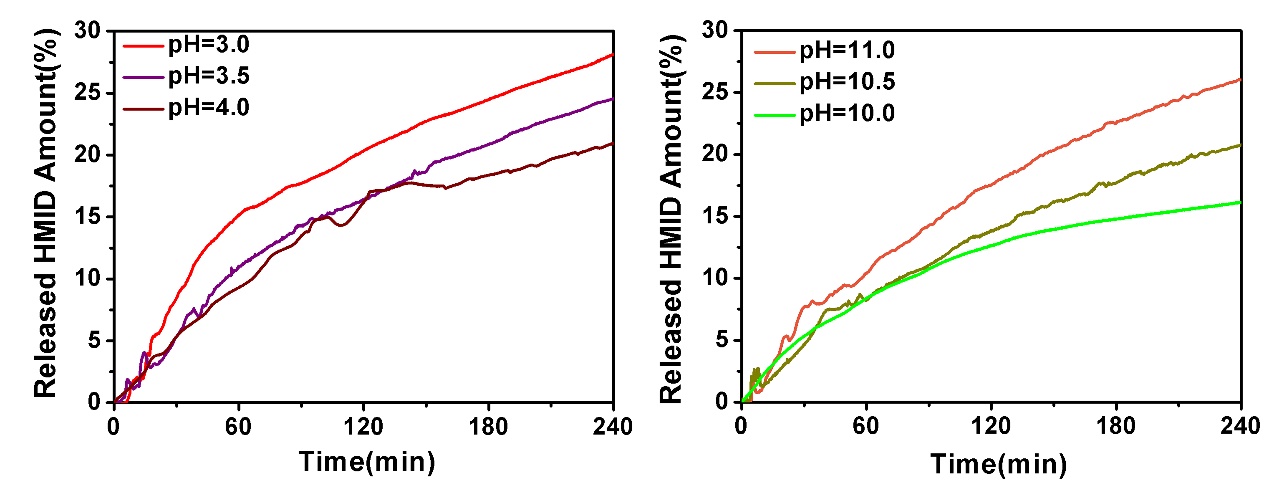
**

**Fig. S3.** Release profiles of HMID from SiO_2_-IMI under different pH values.

**Table S1.** Percentage of HMID in the supernatant from SiO_2_-IMI after being immersed in the solution with different pH values for 4 h.

| pH values | HMID (%) | pH values | HMID (%) |
| --- | --- | --- | --- |
| 3.0 | 28 | 11.0 | 26 |
| 3.5 | 25 | 10.5 | 21 |
| 4.0 | 21 | 10.0 | 16 |

###

###
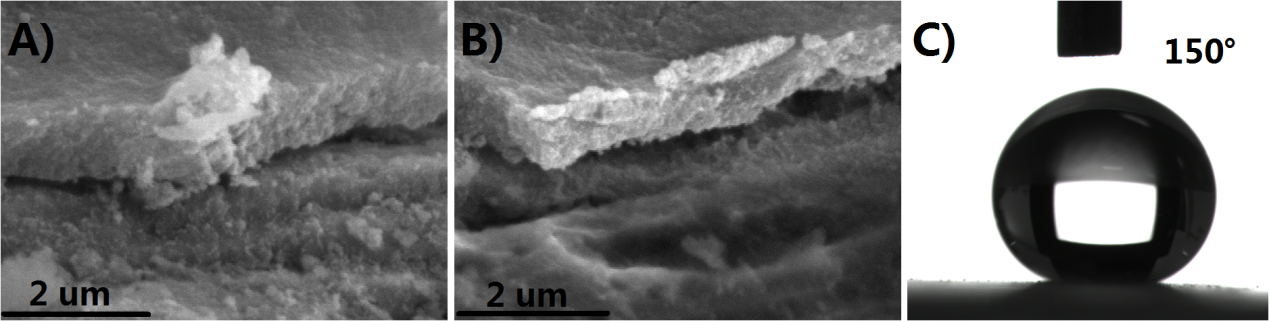
2.4 SEM and CA images

**Fig. S4.** SEM images of the cross section of SHSC (A) and SC (B), and CA image of SHSC (C).

### 2.5 Tafel plots


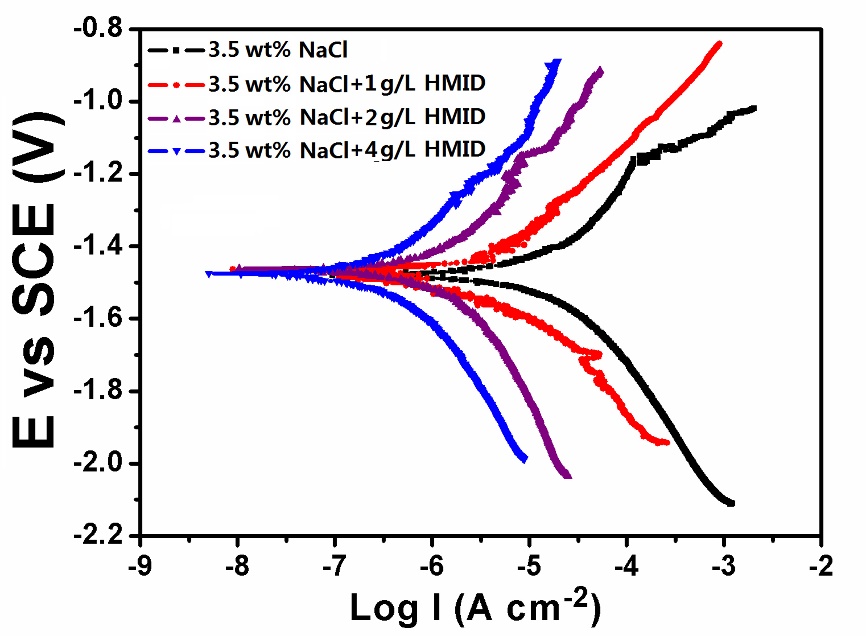


**Fig. S5.** Tafel plots for AA2024 in 3.5 wt% NaCl solution containing different concentration of HMID at 25℃.

| Solution | Inhibitor Concentration, HMID  g/L | *E*_corr_  (mV/SCE) | *I*_corr_  (μA cm^-2^) | *η*_p_  (%) |
| --- | --- | --- | --- | --- |
| 3.5 wt% NaCl | blank | -1482 | 8.51 | — |
|  | 1.0 | -1475 | 2.88 | 66.2 |
|  | 2.0 | -1473 | 1.02 | 88.0 |
|  | 4.0 | -1484 | 0.43 | 94.9 |

**Table S2.** Electrochemical parameters and inhibition efficiencies for AA2024 in 3.5 wt% NaCl solutions containing different concentrations of HMID at 25℃.

The inhibition efficiency, *η*_p_ (%), was obtained by extrapolating the Tafel lines to the corrosion potential by using the following equation:

Where *I*_corr_ and *I*_corr,blank_ are corrosion current densities in the absence and presence of corrosion inhibitors, HMID, respectively.

### 2.6 Laser Micro-Raman spectra


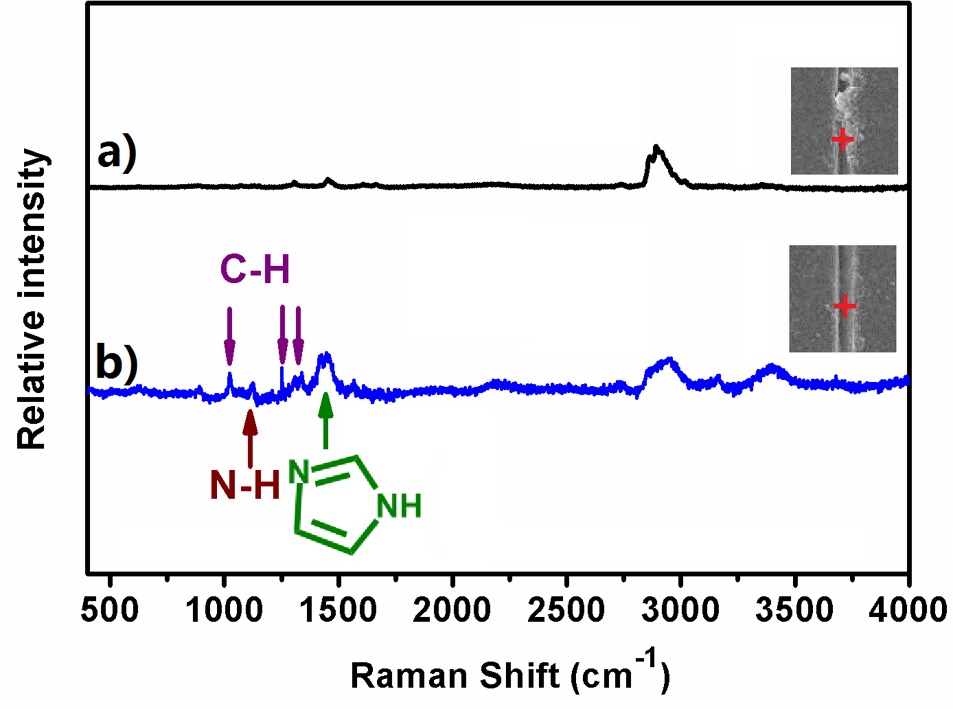


**Fig. S6.** Laser Micro-Raman spectra for artificial scratched region of SHSC (a) and SiO_2_-IMI@SHSC (b).

The artificial scratch (4mm long, 50 μm width, and deep to AA2024 surface) were made on SHSC and SiO_2_-IMI@SHSC by a razor blade. After immersion in 0.1 M NaCl solution for 96 h, the scratches were carefully observed by Laser Confocal inVia Raman Microspectrometer. The corrosion products were clearly seen in the scratch of SHSC. In contrast, the characteristic peaks at 1021, 1254 and 1341 cm^-1^, corresponding to C-H in-plane bending vibration, the peak at 1124 cm^-1^, belonging to N-H in-plane bending vibration, as well as the peak at 1446 cm^-1^ of skeleton vibration of imidazole ring prove the formation of HMID molecular film on the scratch of SiO_2_-IMI@SHSC, which is the main reason for the absence of corrosion products.
